# 

**Published:** 1916-01-29

**Authors:** 


### Buildings Contemplated.

GALWAY.—The Sanatorium Committee of the Galway County Council invite tenders for the erection and completion of the sanatorium at Ryehill, near Athenry, Co. Galway, comprising main block, laundry, engine and pump houses. The architect is Mr. A. Scott, A.R.I.B.A., F.R.I.A.I., 45 Mountjoy Square, Dublin.

HADLEY.—At a meeting of the Shropshire County Council it was stated that the Local Government Board had intimated that they approved the site for an isolation hospital at Hadley, but were not at present able to sanction a loan.

LOUGHBOROUGH.—Funds are being raised to provide an annexe to the Loughborough Hospital. The extension, which is estimated to cost £200, will have a glass roof and glass sides, and will serve both as a dining and recreation room.

### PASSING EVENTS AND TOPICS.

### Pensioners Live Long.

In the first week of the year 1916 the Royal Hospital for Incurables, Putney Heath, lost by death three pensioners: Miss Maria Bowman, elected 1862 (fifty-four years a pensioner); Miss Selina Welcher, elected 1879 (thirty-seven years); and Miss Fanny Williamson, elected in 1882 (thirty-four years).

### Presentation to a Hospital Director.

Mr. F. W. Drewett, Director of the Prince of Wales's General Hospital, Tottenham, was last week presented with his portrait, painted by Mrs. Beavan, a chiming-clock, and an album, the money for which had been raised by public subscription. The presentation was made by Mr. J. Richardson, the vice-chairman.

### The Medical Register.

The number of medical practitioners whose names were added to the Medical Register in 1915 was 1,526. The annual average number added during the five years 1910-14 was 1,172. The number of medical students registered last year was 1,918, while the annual average during the five years 1910-14 was 1,441.

### Games for Invalided Soldiers.

The Brotherhood of St. Andrew in England have appointed a committee to collect and distribute among hospitals and convalescent homes where sick and wounded soldiers are under treatment such articles as dominoes, cards, bagatelle tables, and, in fact, any suitable indoor games. The gifts should be sent to the Games Committee, 17 Cockspur Street, London, S.W.

### Dentists in the French Army.

According to the Paris correspondent of the *Morning Post*, the French Minister of War has arranged that qualified dentists should be given military rank, and be utilised according to their scientific qualifications. A Bill has been brought before the Chamber providing for the creation of a corps of a thousand military dentists, with the rank of adjutant, and a special badge designating their function.

### The Heritage Craft Schools

The Heritage Craft Schools, Chailey, Sussex, have since the war suffered serious financial disabilities. There has been an unavoidable increase in expenditure and a corresponding decrease in income. Under these circumstances it is sincerely hoped that the efforts which are being made to raise funds will be successful. Various functions have been arranged, the profits from which will be used for the Princess Louise Military Wards in connection with the London Hospital and work in connection with the war.

### Military Hospitals and Zeppelin Raids.

The renewed activity of German aerial raiders raises again the question of the protection of hospital buildings from such form of attack. At the request of the Officer Commanding the London District, the Fire Brigade Committee of the London County Council have arranged for an officer of the Fire Brigade to co-operate with an officer of the Royal Engineers in surveying the fire-protection arrangements at military hospitals in the London district, on the condition that neither the Council nor the Chief Officer of the Fire Brigade accepts any responsibility in the matter.

### Panel Doctors on Military Service.

At the present time there are 171 practitioners on the London panel on service with His Majesty's Forces, and in the majority of these cases the Insurance Commissioners are satisfied that the arrangements that have been made for the conduct of their practices during their absence are satisfactory. The Commissioners have recently issued a circular-letter on the subject of medical recruiting, in which they point out that the liability of a panel practitioner under his agreement does not cease when he is called up for service, and that it is only by making arrangements for its fulfilment during his absence that a doctor who goes on service can remain on the panel and have the remuneration accruing under his agreement secured to him.

### What Unopposed M.P.s Might Do.

Mr. James Hill, J.P., the newly-elected Member of Parliament for the Central Division of Bradford, is the hon. treasurer of the Bradford Royal Infirmary. During Mr. Hill's year of office (1908-09) as Lord Mayor of Bradford, and under his auspices, the scheme for the provision of a new Royal Infirmary was inaugurated, and it was largely owing to his example and deep interest in the matter that the sum of £80,000 was promised to the building fund. On the day following his election, unopposed, to the House of Commons Mr. Hill sent a cheque for £400 as a donation to the funds of the infirmary. Mr. Hill has given altogether one thousand guineas (the amount of the cost incurred by a Parliamentary candidate in a contested election), the balance of £650 being distributed amongst eleven other institutions.

### Guy's Hospital Anæsthetics and Operations in 1915.

The number of cases in which general anæsthetics were administered at Guy's Hospital in 1915 was 7,690, among which were sixty-nine intratracheal insufflations of ether, and one of C.E. Spinal anæsthesia was also employed on forty-three occasions, and on sixty-two occasions spinal analgesia followed by a general anæsthetic. Of the 7,733 administrations 177 were for the purpose of examination, 168 for dressings, and 167 for the extraction of teeth, while in two cases three operations were performed under one anæsthetic, in 123 cases two operations were performed under one anæsthetic, in nine cases an operation and an extraction, in fourteen cases an operation and a dressing, in sixteen cases an operation and an examination. In two cases an anæsthetic was given but no operation performed. The total number of operations, therefore, was 7,385 in wards (Bright Ward excepted), special departments, out-patient departments, and surgeries. There was one case of a death under anæsthesia There have been 6,700 cass of general anæsthesia in the dental department: 6,340 under nitrous oxide gas, 348 under ethyl chloride, 11 under ether, and 1 under A.C.E.

RATES OF SUBSCRIPTION: Twelve months, 6/6; Six months, 4/-; Three months, 2/-, post free to any address in the United Kingdom. Abroad, Twelve months, 8/8; Six months, 5/-; Three months, 2/6, post free.—Address The Subscription Dept., "THE HOSPITAL," The Hospital Building, 28/29 Southampton Street, Strand, London, W.C.

**SEND YOUR ORDER NOW**

FOR THE 1916 EDITION OF

**BURDETT'S HOSPITALS & CHARITIES**

**The YEAR-BOOK OF PHILANTHROPY and HOSPITAL ANNUAL.**

Edited by Sir HENRY BURDETT, K.C.B., K.C.V.O.

This authoritative annual work of reference will be published early in the New Year, and it is advisable to place your order without delay, as owing to the increased costs of production the edition will be limited.

OF ALL BOOKSELLERS, OR OF

**THE SCIENTIFIC PRESS, LTD.,**
**28/29 Southampton St., Strand, London, W.C.**

### NATIONAL RELIEF FUND.

**Treasurer—H.R.H. The Prince of Wales.**

**Will every reader of "THE HOSPITAL" send at least 1s.?**

*To* H.R.H. THE PRINCE OF WALES,
BUCKINGHAM PALACE, LONDON.

*I beg to enclose £ : s. d. as a donation to the National Relief Fund.*

*Name* ..............................

*Address* ..............................

..............................

The envelope containing this coupon need not be stamped

